# The effect of different processing methods of linseed on growth performance, nutrient digestibility, blood parameters and ruminate behaviour of lambs

**DOI:** 10.1002/vms3.1149

**Published:** 2023-04-25

**Authors:** Mostafa Hossein Abadi, Taghi Ghoorchi, Elham Amirteymouri, Mohammadreza Poorghasemi

**Affiliations:** ^1^ Department of Animal and Poultry Nutrition Gorgan University of Agricultural Science and Natural Resources Gorgan Iran; ^2^ Department of Animal Science Shahid Bahonar University of Kerman Kerman Iran; ^3^ Department of Animal Science Faculty of Agriculture Rasht Branch Islamic Azad University Rasht Iran

**Keywords:** lambs, linseed, performance, processing, ruminate

## Abstract

**Background:**

Oilseeds such as linseed, canola and sunflower contain unsaturated fatty acids that play important functions in the body. The aim of this study was to assess the effects of different levels of processing linseed on growth performance, nutrient digestibility, blood parameters and ruminate behaviour of lambs.

**Methods:**

Fifty‐six Moghani male lambs (3 months of age, initial average body weight = 28 ± 1.2 kg) were allocated to seven experimental diets in randomized design (eight lambs per each treatment). The experimental diets were as follows: (1) control diet (without linseed), (2) 5% raw linseed, (3) 10% raw linseed, (4) 5% micronized linseed, (5) 10% micronized linseed, (6) 5% extruded linseed and (7) 10% extruded linseed. Lambs were fed ad libitum a basal diet as total mixed ration consisting of 25% concentrate and 75% hay.

**Results:**

The results showed that linseed level and processing method had no significant effect on dry matter intake. Average daily gain, final body weight and feed conversion ratio (FCR) in lambs were affected by experimental diets. The use of 10% micronized linseed and 10% of extruded linseed in the lambs’ diet improved dry matter and crude protein digestibility significantly (*p* < 0.001). Blood glucose concentration observed for lambs fed 10% of micronized or extruded linseed (LS) was not different from that observed in other groups, only from the values shown by lambs fed diets 1 (control) and 2 (5% raw LS). The lowest cholesterol and the highest blood urea nitrogen concentrations were related to lambs fed the control diet (*p* < 0.001). Feeding processed linseed relative to control diet had no effect on feeding behaviour in lambs.

**Conclusion:**

Results of this research showed that the use of extruded and micronized linseed at the level of 10% can improve FCR, nutrient digestibility, and blood parameters.

## Graphical abstract

The aim of this study was to assess the effects of different levels of linseed processing on growth performance, nutrient digestibility, blood parameters and ruminating behaviour of lambs. The results suggest that the use of processed linseed at the level of 10% improves feed conversion ratio, nutrient digestibility and blood parameters. Therefore, extruded and micronized linseed at the level of 10% can be used as a source of energy and protein in lambs diets.

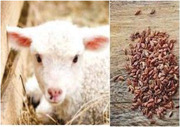

## 1 INTRODUCTION

Current nutritional guidelines suggest that fat consumption should be reduced and that intake of polyunsaturated fatty acids (PUFAs) should be increased compared to saturated fatty acids (Demeyer & Doreau, 1999). Oilseeds such as linseed, canola and sunflower contain unsaturated fatty acids (UFAs), which play important functions in the body (Chichlowski et al., 2005). Linseed contains about 40.5% fat (ether extract), 22% crude protein (CP) and 4% ash and has a very healthy fatty acid profile among oilseeds. Linseed oil contains 9% saturated, 18% monounsaturated and 73% of PUFA (Newkirk, 2008), with the PUFA exerting a great inhibitory effect on methanogens and protozoa (Abuelfatah et al., 2016; Harfoot et al., 1973).

The negative effects of UFAs on rumen fermentation are very important in the nutrition of oilseeds. It has been reported that the increase in the level of UFAs in the rumen leads to a disturbance in fermentation, stopping the growth of different groups of microorganisms, and finally decreasing digestion in the rumen (Kholif et al., 2018).

Research has shown that in ruminants, 87% of alpha‐linolenic intake is biohydrogenated in the rumen before absorption in the small intestine (Dubois et al., 2007). In addition, the addition of UFAs to the diet causes the production of many intermediate materials during the rumen biohydrogenation process that can affect the rumen microbial ecosystem (Glasser et al., 2008). Therefore, the efficiency of the transfer of alpha‐linolenic acid to the duodenum depends on the amount of alpha‐linolenic acid that is protected against the rumen biohydrogenation process (Jenkins et al., 2008).

Various methods are used to process and improve the digestion process of linseeds, and we can mention methods such as extrusion and micronization (Gonthier et al., 2004). The extrusion process is the material processing at high temperature in a short time and is done by the combined action of moisture, heat, mechanical energy and pressure (Gonthier et al., 2005).

Micronization is the processing of oilseeds using infrared radiation (with a wavelength of 1.8–3.4 microns).

This type of radiation works very effectively to heat the oil seeds and by penetrating the oil seeds, increases the molecular movement, which in turn quickly increases the internal temperature (Cheraghi‐Kamalan et al., 2019). Micronization of oilseeds affects their degradability in the digestive system of ruminants. Reducing the degradability of the protein‐rich matrix surrounding fat can be effective in reducing the effects of microorganisms on oilseed lipids (Wang et al., 1997).

Because of the inhibition of ruminal fermentation and potent antimicrobial effects of dietary UFAs, processing of oilseeds such as extrusion leads to altered ruminal fermentation patterns and biohydrogenation pathways (Doreau et al., 2009; Jenkins et al., 2008; Sterk et al., 2010). Feeding extruded linseed to dairy cows increased the proportions of human health‐beneficial omega‐3 fatty acids in milk (Meignan et al., 2017). In another study, Tarricone et al. (2019) reported that bulls fed extruded linseed had significantly greater final and slaughter weights.

These researchers stated that the reason for weight gain due to the use of extruded linseeds is the improvement of the digestibility of nutrients.

In addition, it has been stated in other reports that the feeding of extruded linseeds compared to full‐fat soybean will have higher feed efficiency and weight gain in ruminants (Farran et al., 2008).

Machmüller et al. (2000) concluded in their research that the use of processed linseeds in the diet of fattening lambs can affect the performance of the growth of fattening lambs via reducing methane production, preventing waste of (gas) energy and using the energy to increase weight gain.

Ponnampalam et al. (2015) stated that the increase in yield and improvement in mean weight gain when adding linseed supplements to the diet of fattening lambs could be due to the increase in the consumption of fat as an energy source in the feed.

In another study, it was reported that the consumption of 12% linseed meal in the diet of fattening calves did not have negative effects on the growth performance of calves (Spolare et al., 2005).

Ababakri et al. (2021) showed in their results that diets containing 10% of extruded linseeds, despite their low digestibility, cause higher microbial protein production in ruminants. They stated that from the economic point of view as well as the performance of the diets, using a diet containing 10% of extruded linseeds can be useful and effective.

Bailoni et al. (2004) reported that extruding and roasting of soybean had no effect on rumen fermentation parameters in cows. Inclusion of raw, roasted and extruded soybean in Holstein calves’ diet did not affect the dry matter (DM) intake or performance (Cheraghi‐Kamalan et al., 2019). Lashkari et al. (2018) examined the effects of various linseed oil processing methods on dairy cattle performance and fatty acid patterns and found that experimental diets had no effect on DM intake or milk production. Milk production and DM intake of dairy cattle were not influenced by extruded linseed oil (Samadi et al., 2018).

Tarricone et al. (2019) announced in their reports that dietary supplementation with 3% extruded linseed (EL) improved the amount of n‐3 fatty acids in the meat from young Podolian bulls without affecting their performance.

Gonthier et al. (2004) showed that extrusion is ineffective for protecting linseed protein from rumen degradability, whereas micronizing increases ruminal undegradable protein. Extruded linseed had a negative impact on milk production and milk composition when compared to unprocessed or micronized linseed (Gonthier et al., 2004).

On the other hand, the same researchers, by comparing heat processing of linseeds with extrusion and micronization methods, reported that the increase in digestibility as a result of extrusion heat treatment did not decrease rumen digestibility (Gonthier et al., 2004).

Mustafa et al. (2002) showed that micronization reduces the degradability and increases the post digestibility of linseed in calves. In addition, they showed that micronization is an effective heat treatment to improve the postruminal supply of amino acids from flaxseed.

Petit et al. (2004) also stated in a study that micronization can be used to increase the undegradable protein content in the rumen.

Since little information is available on the simultaneous examination and comparison of the effects of feeding different forms and levels of flaxseed (raw, micronized and extruded) in the diet of lambs, this study was conducted to investigate the effect of different levels of processed linseed in lambs’ diet on performance, nutrient digestibility, blood parameters and ruminate behaviour.

## 2 MATERIALS AND METHODS

### 2.1 Animals and experimental diets

This study was conducted during autumn and winter of 2021. Fifty‐six Moghani male lambs (3 months old) with initial body weight (BW) of 28 ± 1.2 kg were randomly assigned to seven experimental treatments (eight lambs per treatment). Experimental diets were as follows: (1) control diet (without linseed), (2) diet containing 5% linseed, (3) diet containing 10% linseed, (4) diet containing 5% micronized linseed, (5) diet containing 10% micronized linseed, (6) diet containing 5% extruded linseed and (7) diet containing 10% extruded linseed. The study was conducted for 98 days, which included 14 days of adaptation period and 84 days for data recording. The animals were kept in individual stalls and had free access to water during the experimental period. The diet of the lambs in this experiment was adjusted based on BW and according to the nutritional requirements tables of small ruminants (NRC, 2007). The components of the diet are shown in Table 1. The experimental diets were fed to lambs as total mixed ration (TMR; consisting of 25% concentrate and 75% hay) and ad libitum at 8:00 AM and 4:00 PM. The hay used was of a local type (Qara alfalfa) in chopped form and was 2–3 cm in size, and the concentrate was in powder form. Orts were recorded and weighed every day. All procedures related to animals were certified by the Animal Care described by the Iranian Council of Animal Care (ICAC, 1995). The lambs were shorn and treated with albendazole (Roacel) for internal parasites.

**TABLE 1:** The ingredients and chemical analysis of experimental diets (% of DM)

	Experimental diets^a^						
Ingredients	1	2	3	4	5	6	7
Alfalfa hay, chopped	30	30	30	30	30	30	30
Wheat straw, chopped	10	10	10	10	10	10	10
Barley grain, ground	29	26	23	26	23	26	23
Corn grain, ground	10	10	10	10	10	10	10
Soybean meal	11	9	7	9	7	9	7
Raw linseed	0	5	10	0	0	0	0
Micronized linseed	0	0	0	5	10	0	0
Extruded linseed	0	0	0	0	0	5	10
Wheat bran	7	7	7	7	7	7	7
Vitamins A, D and E premix^a^	1	1	1	1	1	1	1
Trace‐mineralized salt^b^	0.5	0.5	0.5	0.5	0.5	0.5	0.5
Sodium bicarbonate	0.5	0.5	0.5	0.5	0.5	0.5	0.5
Limestone	1	1	1	1	1	1	1

Chemical composition							
DM (%)	87.42	87.42	87.42	87.42	87.42	87.42	87.42
Crude protein (% DM)	12	12	12	12	12	12	12
EE (% DM)	3.83	5.38	5.67	5.81	5.53	5.42	5.74
NDF_om_ (%)	37.68	37.91	38.15	37.91	38.15	37.91	38.15
ADF_om_ (%)	23.84	23.98	24.11	23.98	24.11	23.98	24.11

Abbreviations: ADF, acid detergent fiber; DM, dry matter; EE, ether extracts; NDF, neutral detergent fiber; OM, Organic matter.

^a^ 1: Control without linseed; 2: 5% raw linseed; 3: 10% raw linseed; 4: 5% micronized linseed; 5: 10% micronized linseed; 6: 5% extruded linseed; 7: 10% extruded linseed.

### 2.2 Performance

DM intake was calculated as the difference between the weight of the feed provided and ort. On average, 1.5 kg of feed was provided for each lamb every day. The lambs were weighed at the beginning of experiment and every 14 days until the end of the experiment for calculating average daily gain and feed conversion ratio (FCR).

The weight of the lambs was measured throughout the experiment period using a Satrue livestock digital scale with a capacity of 300 kg (Satrue, MAX‐P300, Taiwan) and an accuracy of 100 g.

The TMR samples were collected weekly, and DM was measured at 60°C for 48 h (AOAC, 2002). The lambs FCR was calculated by dividing DM intake by average daily gain.

### 2.3 Digestibility

The internal marker of acid‐insoluble ash was used to measure the apparent digestibility of nutrients. Thirty‐five lambs (five lambs for each treatment) were used for faeces sampling to determine nutrient digestibility in the last 3 days of the experiment. A sampling of faeces was performed directly through the rectum twice a day with an interval of 3 h (the first one was taken about 4 h after consuming feed). The daily collected faeces samples of each lamb were mixed, and finally a 100‐g sample of faeces was packed in a plastic bag and stored at −20°C for chemical analysis. Nutrient digestibility was calculated using the relationship proposed by Church and Pound (Van Keulen & Young, 1977):

$$\mathrm{AD}\left(\%\right)=100-100\times \left(\mathrm{MD}/\mathrm{MF}\right)\times \left(\mathrm{NF}/\mathrm{ND}\right),$$

where AD is the apparent digestibility (%), MD is the marker in the diet (%), MF is the marker in the faeces (%), NF is the nutrient in the faeces (%) and ND is the nutrient in the diet (%).

Chemical composition of diet and faeces including crude protein (Micro Kjeldahl, Foss 2010, Sweden), the insoluble fibre in neutral detergent (Van Soest et al., 1991), crude fat from Soxhlet apparatus (Behr. Labor‐Technik), DM and insoluble fibre in acid detergent as well as organic matter were measured according to standard methods (AOAC, 2002).

### 2.4 Ruminal parameters

For the evaluation of ruminal factors, the ruminal contents were collected from the rumen on the 80th day of period, 3 h after the morning meal through the mouth using an oesophageal tube and a vacuum pump. The ruminal pH was monitored immediately after the sample collection with a pH meter (AZ, model 8689). The ruminal contents were strained through three layers of cheesecloth, and 15 mL ruminal fluid was mixed by 3 mL of 0.2N HCL and frozen at −20°C to determine the ammonia nitrogen concentration (Broderick & Kang, 1980).

To measure rumen volatile fatty acid (VFA) concentration, 4 mL rumen fluid samples were mixed with H_3_PO_4_. Gas chromatography equipment (Bayatkouhsar et al., 2022) was used to analyze the volatile fatty acids of the samples. 2‐Ethylbutyric acid was also used as an internal standard for injection into the gas chromatograph. Before injecting into the device, the samples were first centrifuged for 15 min at 15,000 × *g* at a temperature of 4°C and then the clear supernatant liquid was transferred into special chromatography vials. For each sample, an amount of 2 μL was injected into the device using a Hamilton syringe. The column of the device (Carboxen TM 1000, 60/45) was 122 cm long and 1.8 mL in diameter. The flow of nitrogen, hydrogen, and air into the column was 30, 30, and 320 mL/min, respectively (Lee et al., 2005).

### 2.5 Blood parameters

On the last day of the experiment, 10 mL blood samples were taken from the jugular vein of the lambs to measure the blood parameters. The blood samples were transferred into tubes without heparin and then the tubes were quickly sent to the laboratory in a flask containing ice. The tubes were centrifuged for 10 min at 3000 rpm to separate the serum. Glucose, triglyceride, cholesterol and blood urea nitrogen (BUN) contents of the samples were measured by Pars Azmoon commercial kits, using a BT 1500 auto‐analyzer (Rome, Italy).

### 2.6 Feeding behaviour

The feeding behaviour of the lambs was evaluated over 24 h (8 AM on the 83rd day to 8 AM on the 84th day of the experiment) and at 5‐min intervals by digital cameras (720P HD IP Camera; SecuEasy, Korea). The investigated variables included duration of eating, ruminating, chewing and resting. If a lamb was eating diet while standing next to the diet buckets, it was considered an eating activity, and if it was chewing while resting or away from the diet buckets, it was considered a rumination activity. The total time spent eating and ruminating was used for the chewing activity. In addition, the length of time the lambs were sitting or sleeping (without chewing activity) was used for the resting variable.

### 2.7 Statistical analysis

The obtained data were analyzed based on a completely randomized design with seven treatments and eight replicates, using the SAS statistical software version 9.1 (2001) and the general linear model. In addition, the average treatments were compared through Duncan's test at a significance level of 5%.

## 3 RESULTS

The linseed level and method of processing do not have a significant effect on DM intake. Average daily gain (ADG), final body weight (FBW) and FCR in lambs were affected by the experimental diets. ADG and FBW were greater in lambs fed diets containing 10% extruded linseed and 10% micronized linseed (*p* < 0.01), while these lambs had significantly lower FCR (*p* < 0.001) than the lambs fed with other experimental diets (Table 2). Results showed that DM, CP and EE digestibility were influenced by the experimental diets (*p* < 0.05). Generally, 10% extruded and micronized linseed improved the nutrients’ digestibility. OM, NDF and ADF digestibility were not affected by treatments (*p* > 0.05; Table 3). Mean rumen fluid pH, VFAs and TVFA concentration were not changed by the processing of linseed (*p* > 0.05). NH_3_ concentration did not decrease with increasing micronized linseed (LS) but decreased with 10% extruded LS (*p* < 0.01; Table 4). Blood glucose concentration observed for lambs fed 10% of micronized or extruded LS was not different from that observed in other groups, only from the values shown by lambs fed diets control and 5% raw LS (*p* < 0.01). The triglyceride level of the lambs that used 10% processed linseeds (micronized and extruded) increased significantly only compared to the control (*p* < 0.05). The cholesterol level of lambs that were fed with 10% processed linseeds (micronized and extruded) had a significant increase compared to the control and the treatment containing 5% raw linseeds (*p* < 0.05). The amount of urea nitrogen in lambs that were fed with 10% micronized or extruded linseeds (5% and 10%) had a significant decrease compared to the control and other treatments (*p* < 0.001; Table 5).

**TABLE 2:** Effects of processing method and level of linseed on performance

Experimental diets^a^									
Parameter	1	2	3	4	5	6	7	SEM	*p*‐Value
Dry matter intake (g/day)	1242.8	1249.9	1248.1	1259.9	1272.6	1264.6	1280	12.92	0.6896
Average daily gain (g/day)	218.9^d^	221.4^d^	220.5^d^	226.4^cd^	249.38^b^	231^c^	260.9^a^	2.94	0.0001
Final body weight (kg)	45.62^d^	46.47^bdc^	46.29^dc^	47.32^abc^	47.66^ab^	47.52^abc^	48.41^a^	4.05	0.0023
Feed conversion ratio	5.7^a^	5.8^a^	5.7^a^	5.6^a^	5.1^b^	5.5^a^	4.9^b^	0.09	0.0001

*Note*: Values with differing letters within the same column are significantly different (*p* < 0.05).

Abbreviation: SEM, standard error of the mean.

^a^ 1: Control without linseed; 2: 5% raw linseed; 3: 10% raw linseed; 4: 5% micronized linseed; 5: 10% micronized linseed; 6: 5% extruded linseed; 7: 10% extruded linseed.

**TABLE 3:** The effect of different levels and processing of linseed on nutrient digestibility (%)

Experimental diets^a^									
Parameters	1	2	3	4	5	6	7	SEM	*p*‐Value
Dry matter	66.1^c^	70.9^b^	71.5^b^	71.4^b^	77.5^a^	76.6^b^	79.6^a^	1.14	0.0001
Organic matter	68.3	70.4	73.5	70.8	72.9	69.1	68.5	1.92	0.2846
Crude protein	60.5^b^	62.1^b^	62.6^b^	64.5^b^	70.9^a^	64^b^	74.1^a^	1.45	0.0001
Neutral detergent fibre	58.9	58.4	57.3	59	57.5	61.9	57	1.91	0.4288
Acid detergent fibre	49.4	51.3	52.7	50.9	49.3	53.2	50.4	1.38	0.5422
Crude fat	59.9^c^	63.4^b^	64.4^ab^	64.9^ab^	65.9^ab^	66.1^ab^	67.1^a^	0.90	0.0001

*Note*: Values with differing letters within the same column are significantly different (*p* < 0.05).

Abbreviation: SEM, standard error of the mean.

^a^ 1: Control without linseed; 2: 5% raw linseed; 3: 10% raw linseed, 4: 5% micronized linseed; 5: 10% micronized linseed; 6: 5% extruded linseed; 7: 10% extruded linseed.

**TABLE 4:** The effect of different levels and processing of linseed on rumen parameters (%)

Experimental diets^a^									
Rumen fermentation profile	1	2	3	4	5	6	7	SEM	*p*‐Value
NH_3_ mg/dL	15.30^b^	15.52^ab^	15.92^ab^	14.79^bc^	16.37^a^	15.68^ab^	14.17^c^	0.34	0.0056
pH	6.25	6.19	6.37	6.27	6.20	6.25	6.28	0.09	0.86
Total volatile fatty acids (mmol/L)	107.31	105.83	107.82	106.01	107.23	109.03	106.40	5.07	0.99
Volatile fatty acids									
Acetate Mm/100 mM	61.18	59.04	62.13	58.29	61.29	59.99	58.97	3.01	0.96
Propionate Mm/100 mM	26.45	25.81	24.68	25.85	24.03	25.72	25.53	2.51	0.98
Isobutyrate Mm/100 mM	3.08	2.30	2.31	2.63	2.81	3.09	3.33	0.81	0.95
Butyrate Mm/100 mM	9.12	10.39	10.15	10.56	10.23	10.67	10.95	0.85	0.81
Isovaleric acid Mm/100 mM	5.78	5.95	5.50	5.86	5.99	6.02	5.11	1.37	0.99
Valeric acid Mm/100 mM	2.51	2.32	3.04	2.80	2.86	2.51	2.48	0.67	0.98
Acetate/propionate Mm/100 mM	2.66	2.29	2.54	2.45	2.60	2.25	2.33	0.33	0.96

*Note*: Values with differing letters within the same column are significantly different (*p* < 0.05).

Abbreviation: SEM, standard error of the mean.

^a^ 1: Control without linseed; 2: 5% raw linseed; 3: 10% raw linseed; 4: 5% micronized linseed; 5: 10% micronized linseed; 6: 5% extruded linseed; 7: 10% extruded linseed.

**TABLE 5:** Blood parameters of lambs fed diets containing different levels and processing of linseed

Parameters (mg/dL)	Experimental diets^a^								
	1	2	3	4	5	6	7	SEM	*p*‐Value
Glucose	59.1c	60.5^bc^	62.6^ab^	63.8^ab^	64.1^a^	64.8^a^	64.5^a^	1.15	0.0037
Triglyceride	26.8^b^	28.4^ab^	28.1^ab^	29.8^ab^	30.5^a^	29.3^ab^	31.4^a^	1.17	0.0253
Cholesterol	59.4^d^	61.1^cd^	62.1^bcd^	64.5^abc^	65.9^ab^	63.8^abc^	66.3^a^	1.29	0.0291
Urea nitrogen	15.9^a^	13.9^b^	14.2^b^	13.4^b^	11.9^c^	11.3^c^	10.7^c^	0.44	0.0001

*Note*: Values with differing letters within the same column are significantly different (*p* < 0.05).

Abbreviation: SEM, standard error of the mean.

^a^ 1: Control without linseed; 2: 5% raw linseed; 3: 10% raw linseed; 4: 5% micronized linseed; 5: 10% micronized linseed; 6: 5% extruded linseed; 7: 10% extruded linseed.

Feeding behaviour was not affected by using processed linseed in the lambs’ diet (*p* > 0.05) (Table 6).

**TABLE 6:** The effect of using linseed processing and different levels on feeding behaviour

	Experimental diets^a^								
Treat (min/day)	1	2	3	4	5	6	7	SEM	*p*‐Value
Eat	361	354.38	370.13	366.75	353.13	350.50	361.25	8.67	0.5840
Rumination	262.75	257.88	240.38	244.88	264.38	259.88	221.50	13.78	0.3094
Chew	623.75	612.25	610.50	611.63	617.50	610.38	582.75	12.68	0.6006
Rest	816.25	827.75	829.50	828.38	822.50	829.63	857.25	12.68	0.6006

Abbreviation: SEM, standard error of the mean.

^a^ 1: Control without linseed; 2: 5% raw linseed; 3: 10% raw linseed; 4: 5% micronized linseed; 5: 10% micronized linseed; 6: 5% extruded linseed; 7: 10% extruded linseed.

## 4 DISCUSSION

The differences in DM intake were not significant in any of the treatments. It has been suggested that linseed processing increased palatability and thus improved DM intake. In our previous study, different levels of linseed did not have a significant effect on DM intake of pre‐weaning calves (Hossein Abadi et al., 2020). In agreement with our data, Cheraghi‐Kamalan et al. (2019) reported that feeding diets containing soybean oil, roasted soybean and extruded soybean had no effect on DM intake in steers. Gonthier et al. (2004) also found that DM and organic matter intake were not affected by linseed and its heat processing supplementation. In contrast to our results, Amirteymouri et al. (2021) reported that DM intake in lambs fed ground linseed was higher than in lambs that received extruded linseed.

In the current study, lambs fed diets containing extruded and micronized linseeds at a level of 10% had higher ADG, FBW and lower FCR compared to other groups, and these differences were due to differences in digestible DM intake. Digestible DM intake influences average daily gain and improves livestock performance (Mc Donald et al., 2002). In other words, improvement of the FCR in the extruded and micronized treatments may be associated with better consumption of crude protein. The process of micronization and extrusion causes the deactivation of anti‐nutritional substances, and following this the availability of protein increases and improves the animal growth (Alonso et al., 2000). Acuti et al. (2012) investigated the effects of extruded linseed and conjugated linoleic acid on the growth performance of suckling calves and reported that feeding extruded linseed improved the ADG and final weight of the calves. Extruded and roasted soybeans in lambs’ diet increased and decreased ADG and FCR, respectively (Aliyari et al., 2018). Oilseed processing methods such as milling, roasting and extrusion have altered rumen fermentation parameters and livestock performance due to the effect of these processing on oil and protein availability in these seeds (Faldet & Satter, 1991).

DM, crude protein and EE digestibility were affected by experimental diets in this study. The inclusion of processed linseed in the experimental diets improved nutrients’ digestibility. The lambs that received a treatment diet containing 10% extruded and micronized linseed had higher apparent DM and EE digestibility in the whole digestive tract than those fed other experimental diets. Likely, heat processing of oilseeds alters the protein digestion place from rumen to small intestine and improves total tract nutrient digestibility (Wang et al., 1997). Unlike other heating processes, micronization method generates heat from inside the seed. Micronization increases pressure inside the oilseeds and cracks the seed coat, which prevents ruminal microorganisms’ access to internal seed components for digestion (Wang et al., 1997). Gonthier et al. (2004) and Martin et al. (2007) did not find any effect on nutrient digestibility with extruded linseeds. Linseed in our previous study on pre‐weaning calves increased DM digestibility (Hossein Abadi et al., 2020). Moreover, linseed at 5% significantly improved the digestibility of ether extract. Pires et al. (1997) noted that the digestibility of organic matter was increased by ground cottonseed, while Lashkari et al. (2018) showed digestibility of DM, organic matter and crude protein were not influenced by processed and unprocessed linseeds. Nonsignificant changes in lambs NDF digestibility were consistent with the study of Giannico et al. (2009) using rolled and ground linseeds in lambs. In Gonthier et al.’s (2004) study, total tract NDF and ADF digestibilities were similar for micronized and extruded linseeds. Other studies also showed no effect of feeding heat‐treated oilseeds on total tract fibre digestion (Petit et al., 2001; Scott et al., 1991; Shabi et al., 1999).

As indicated, the experimental diets had no significant effect on the lambs’ ruminate behaviour. The ratio of forage to concentrate was maintained constant in all treatments, and the amount of forage was the same across all groups. The physical characteristics of feed can influence livestock nutrition behaviour and performance. Moreover, ration fibres including forage and non‐forage fibres perform differently in ruminant activity stimulation since those fibres vary in particle size and the residence time in the rumen (Mertens, 1997). In this research, chewing activity, eating behaviour and ruminating time based on minutes per day in all experimental diets that had the same forage to concentrate rate were not affected. In fact, the addition of treated linseed as a high‐fat ingredient in lambs’ diet could not negatively affect fibre digestion. Maekawa et al. (2002) reported that changes in rumination time may be related to differences in DM intake as well as nutrients’ digestibility. Roasted and extruded soybeans or their mixture had no effect on the pattern of eating and or chewing activity in dairy cows (Sadr Erhami et al., 2015). Alyari et al. (2018) noted that feeding lambs with crude, extruded and roasted soybean instead of soybean meal had no effect on feeding behaviours.

The rumen fluid pH and fatty acids concentration were not significantly affected by the experimental diets. However, the concentration of ammonia nitrogen in the rumen fluid decreased significantly when using 10% extruded linseed compared to unprocessed linseed (*p* < 0.01). Ahrar and Schingoethe (1979) reported that rumen ammonia and pH were not affected by soybean meal and heated soybean meal diets. Aldrich et al. (1995), Faldet et al. (1992) and Stern et al. (1985) reported that extruded soybeans compared to raw soybeans reduced degradability of crude protein in the rumen. In Fathi Nasri et al.’s (2008) study, roasting reduced the rumen degradability of soybean crude protein compared to raw soybean, which is consistent with our study.

In this regard, it is stated in the results of some reports that the higher release of fatty acids in a diet containing extruded linseeds affects the permeability of the microbial membrane and important metabolic pathways by creating a coating of lipids on the surface of the microbes that can be used as a regulator of rumen microbial activity to better use ammonia to increase microbial protein synthesis (C. Wang et al., 2018).

In other studies, similar findings have been reported on applying thermal processing to soybean and canola meals. Heat facilitates the Maillard reaction between aldehyde groups of sugars and free amino acid groups of proteins to form amino‐sugar complexes. Compared to natural peptides, this complex is more resistant to enzymatic hydrolysis (Demjanec et al., 1995; Moshtaghinia & Ingalls, 1992). These heat treatments also reduce the soluble part of the feed protein, which reduces the deamination of amino acids and ammonia nitrogen produced in the rumen and finally reduces the breakdown of structural protein into volatile fatty acids such as isobutyric acid, isovaleric acid, valeric acid and acetic acid, and this can be effective in increasing the rate of protein passage and escape from the rumen to the small intestine and increasing microbial protein synthesis (Seifdavati & Taghizadeh, 2012).

It seems that heat processing in this experiment has been effective in reducing access of rumen microorganisms to protein and increasing its passage through the rumen with the changed linseed proteins structure. However, this suggested that the protein fraction in the heat‐treated oilseed meal was degraded at a slower rate in the rumen by the microorganisms than in the unheated meal.

The absence of a difference in all VFAs and TVFA concentration due to extrusion and micronization of linseeds is partly due to the absence of a general effect of processed linseed in this experiment on fibre digestibility (Doreau et al., 2009). In addition, the VFA concentrations, proportions of VFA and protozoa population did not differ among the diets containing extruded and rolled linseed either before or after the morning meal in dairy cows (Doreau et al., 2009).

According to these results, blood glucose, triglyceride and cholesterol concentration were greater (P < 0.05) for lambs fed processed linseed (extrude and micronized) than for those fed the control diet. The higher blood glucose in lambs fed 10% extruded and micronized linseed is related to increasing the digestibility of DM intake in these lambs. A higher digestibility could imply a higher absorption of propionate in the rumen or a higher absorption of glucose in the small intestine.

Studies have shown that fatty acids are biohydrogenated in the rumen, and by changing the fermentation pattern, they increase the amount of propionate compared to acetate. Propionate is the main precursor of gluconeogenesis and leads to the synthesis of glucose. In addition, the glycerol resulting from fat hydrolysis in oilseeds is converted into propionate, which causes an increase in blood glucose levels through gluconeogenesis (Lawrence et al., 2016). In the present experiment, due to the lack of significant increase in the concentration of propionate, the fatty acids of linseeds processed by biohydrogenation were preserved in the rumen, and also due to the increase in the digestibility of crude fat in processed treatments compared to the control, it seems that the glycerol resulting from fat hydrolysis in the intestine is the main source of gluconeogenesis to increase glucose.

Moreover, using processed linseed caused an improved flow of amino acids to the intestine as well as more microbial protein synthesis (Amirteymori et al., 2021), which improves the process of gluconeogenesis and blood glucose concentration (Young, 1977). In Liu et al.’s (2008) study, the plasma glucose was not affected by dietary sources of roasted oilseeds in lactating Holsteins. In another study (Danesh Mesgaran et al., 2012), cows fed a ground linseed diet showed a decrease in blood glucose compared with that of whole linseed and extruded soybean.

Oilseeds as fat supplementation increase the concentration of cholesterol in dairy cows (Delbecchi et al., 2001; Petit et al., 2004). The reason for the increase in cholesterol concentration is fatty acid mobilization (Gonthier et al., 2004). Blood concentration of cholesterol was increased by feeding different kinds of oilseeds to cows (Petit et al., 2004). Plasma cholesterol concentration was greater for cows that received heat‐treated linseed (raw, micronized and extruded) than a diet without linseed (Gonthier et al., 2004). In the present experiment, the differences in BUN could be related to differences in protein requirements as consequence of differences in ME intake and growth rate. In fact, the higher ME intake determines a higher ADG and therefore a higher protein requirement.

## 5 CONCLUSION

Results of this research showed that use of processed linseed at the level of 10% improved FCR, digestibility and blood parameters. So, extruded and micronized linseeds at the level of 10% can be used as a source of energy and protein in lambs’ diets. It is also suggested that in future studies, the effect of feeding these diets on lambs’ performance and their meat composition should be investigated.

## AUTHOR CONTRIBUTIONS

*Conceptualization, investigation, project administration, supervision, writing‐ original draft, and writing‐review and editing*: Mostafa Hossein Abadi. *Conceptualization, data curation, formal analysis, investigation, methodology, project administration, supervision, writing‐ original draft, and writing‐review and editing*: Taghi Ghoorchi. *Data curation, investigation, project administration, writing‐original draft, and writing‐review and editing*: Elham Amirteymouri. *conceptualization, supervision, visualization, and writing‐review and editing*: Mohammadreza Poorghasemi.

## CONFLICT OF INTEREST STATEMENT

The authors declare no conflict of interest.

## FUNDING INFORMATION

This research was done with the personal funding of the authors.

## ETHICS STATEMENT

The experimental protocol was ratified by the Animal Ethic Committee of the Gorgan University of Agricultural Science and Natural Resources, Gorgan, Iran, and the experiment was performed with respect to the International Guidelines for research involving animals (Directive 2010/63/EU).

### PEER REVIEW

The peer review history for this article is available at https://publons.com/publon/10.1002/vms3.1149.

## Data Availability

Availability of data I certify that all data in the article are true and reliable. All public data generated or analyzed during this study are included in this article. Data sharing is not applicable to this article as no new data were created or analyzed in this study.
